# Endoscopic Skipping, Stricturing, and Penetrating Complications in Crohn’s Disease on Tandem Ileo-colonoscopy and Cross-sectional Imaging: A Retrospective Cohort Study

**DOI:** 10.1093/ibd/izae192

**Published:** 2024-08-31

**Authors:** Virginia Solitano, Sudheer Kumar Vuyyuru, Achuthan Aruljothy, Maan Alkhattabi, Joshua Zou, Melanie Beaton, Jamie Gregor, Zahra Kassam, Rocio Sedano, Harry Marshall, Darryl Ramsewak, Michael Sey, Vipul Jairath

**Affiliations:** Division of Gastroenterology, Department of Medicine, Western University Schulich School of Medicine, London, Ontario, Canada; Division of Gastroenterology and Gastrointestinal Endoscopy, IRCCS Ospedale San Raffaele, Università Vita-Salute San Raffaele, Milan, Italy; Division of Gastroenterology, Department of Medicine, Western University Schulich School of Medicine, London, Ontario, Canada; Division of Gastroenterology, Department of Medicine, Western University Schulich School of Medicine, London, Ontario, Canada; Division of Gastroenterology, Department of Medicine, Western University Schulich School of Medicine, London, Ontario, Canada; Department of Medicine, Faculty of Medicine, King Abdulaziz University, Rabigh Campus, Saudi Arabia; Department of Biostatistics, University of Waterloo, Waterloo, Ontario, Canada; Division of Gastroenterology, Department of Medicine, Western University Schulich School of Medicine, London, Ontario, Canada; Division of Gastroenterology, Department of Medicine, Western University Schulich School of Medicine, London, Ontario, Canada; Department of Medical Imaging, Western University, London, Ontario, Canada; Division of Gastroenterology, Department of Medicine, Western University Schulich School of Medicine, London, Ontario, Canada; Department of Epidemiology and Biostatistics, Western University, London, Ontario, Canada; Department of Medical Imaging, Western University, London, Ontario, Canada; Department of Medical Imaging, Western University, London, Ontario, Canada; Division of Gastroenterology, Department of Medicine, Western University Schulich School of Medicine, London, Ontario, Canada; Lawson Health Research Institute, London, Ontario, Canada; Division of Gastroenterology, Department of Medicine, Western University Schulich School of Medicine, London, Ontario, Canada; Department of Medical Imaging, Western University, London, Ontario, Canada

**Keywords:** inflammatory bowel disease, diagnosis, endoscopic skipping, penetrating complications

## Abstract

**Background:**

Crohn’s disease (CD) is characterized by discontinuous inflammation. Failure to identify skipping lesions of the terminal ileum (TI) or transmural changes can lead to incorrect management.

**Methods:**

Eligible adult patients with CD undergoing ileo-colonoscopy and computed tomography enterography or magnetic resonance enterography within 6 months. We determined the prevalence of endoscopic skipping (normal ileum on colonoscopy but proximal small bowel inflammation on cross-sectional imaging), skip lesions (discontinuous inflammation along the gastrointestinal tract identified on cross-sectional imaging), structuring, and penetrating complications.

**Results:**

Among 202 patients, 45 (22.3%) had endoscopic skipping proximal to TI intubation. Fifty patients (24.5%) had small bowel skip lesions, primarily in the ileum. Strictures were identified in 34 patients (16.8%) through both imaging and ileo-colonoscopy, in 21 patients (10.4%) solely through cross-sectional imaging, and in 3 patients (1.5%) solely through ileo-colonoscopy. Approximately 36.2% of stricturing cases would be missed without cross-sectional imaging. Penetrating complications, including abscesses (2.5%) and various fistula types (4.9%), were detected in 15 (7.4%) patients.

**Conclusions:**

Ileo-colonoscopy missed detection of active CD in approximately one-fifth of cases due to more proximal disease location. Stricturing disease might be missed in more than a third of cases if cross-sectional imaging is not performed.

## Graphical Abstract

**Graphical Abstract:**
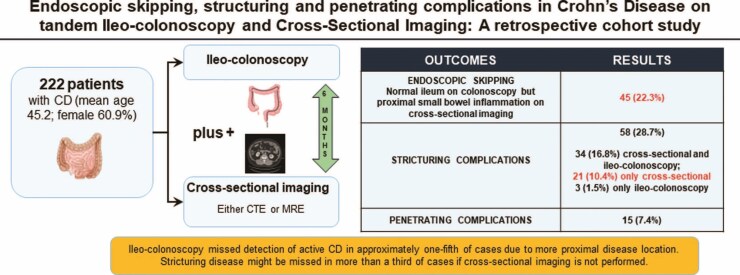

**Key Messages**

**What is already known?**

Crohn’s disease (CD) presents with discontinuous inflammation, but endoscopic assessment may miss proximal lesions.

**What is new here?**

This study reveals that approximately one-fifth of CD cases exhibit endoscopic skipping, with cross-sectional imaging detecting skip lesions and strictures often missed by endoscopy.

**How can this study help patient care?**

Utilizing both endoscopy and cross-sectional imaging can significantly enhance CD management by accurately identifying skip lesions, strictures, and penetrating complications, thereby guiding appropriate treatment strategies.

## Introduction

Crohn’s disease (CD) is a chronic inflammatory bowel disease (IBD), typically characterized by periods of relapse and remission, that can lead to penetrating complications, progressive bowel damage, and surgical resection.^1^ Inflammation can occur anywhere in the gastrointestinal tract, from mouth to the anus, and can be discontinuous in nature.^2^ Disease mapping therefore typically requires cross-sectional imaging alongside ileo-colonoscopy for comprehensive evaluation.^3^ Commonly used methods for whole small bowel evaluation are computerized tomography enterography (CTE) or magnetic resonance enterography (MRE).^4^ These modalities enable the assessment of proximal small bowel inflammation as well as transmural complications, such as strictures, fistulas, or abscesses, which might not be fully discernible via ileo-colonoscopy alone.^5^

The selection of the imaging modality may be influenced by various factors, including patient age, clinical considerations, and local resource availability. Computerized tomography enterography is widely available and has a high spatial resolution, with the main drawback being exposure to ionizing radiation.^6^ Both CTE and MRE are sensitive and specific for identifying strictures throughout the small bowel.^7,8^ Furthermore, they provide crucial additional information that may influence clinical management, including the presence and severity of inflammation, stricture length, upstream bowel dilatation, and other complications, including abscesses or fistulas.^9^ Beyond their diagnostic utility, these imaging techniques play a pivotal role in assessing the response to therapeutic interventions. Well-established radiologic scoring systems (eg, magnetic resonance index of activity [MaRIA], simplified MaRIA, London index, and London extended index), characterized by high interobserver agreement and responsiveness, can assess the effectiveness of treatments in managing CD.^10^

Patients with CD may have inflammation distributed more proximally than the terminal ileum (TI) which is out of reach of the colonoscope, so-called endoscopic skipping of the TI.^11,12^ Data from a retrospective study of pediatric patients with small bowel CD, who may naturally present with a more aggressive phenotype, reported endoscopic skipping of the TI in 59% of patients.^13^ Furthermore, there may be transmural changes of inflammation seen in the TI by imaging despite the absence of mucosal inflammation detected by ileo-colonoscopy. Thus, the failure to detect skipping of the TI or transmural changes may result in delayed or erroneous management of CD (eg, patients might be inadvertently enrolled in clinical trials with proximal small bowel inflammation).

The aim of this study was to determine the prevalence of endoscopic skipping, defined as normal ileum on colonoscopy but proximal small bowel inflammation on cross-sectional imaging, as well as the prevalence of stricturing and penetrating complications in adult patients with CD through a retrospective evaluation of tandem CTE or MRE imaging and ileo-colonoscopy.

## Materials and Methods

### Study Population and Design

This retrospective study included adult patients (aged ≥18 years) with a confirmed and established diagnosis of CD based on standard clinical, endoscopic, and histologic criteria. Eligible participants were patients with CD who had undergone ileo-colonoscopy with terminal ileal intubation as well as cross-sectional imaging (CTE or MRE) within a 6-month timeframe of each other. Both procedures were done as part of routine clinical care between January 1, 2010 and December 31, 2018, at the London Health Sciences Centre and St. Joseph’s Health Centre in London, Canada. Patients with a prior proctocolectomy with an end ileostomy or ileal pouch–anal anastomosis were excluded. The reporting of this study is following the STROBE (Strengthening the Reporting of Observational Studies in Epidemiology) statement.

### Data Collection

An electronic search of radiology databases identified patients with a diagnosis of CD who underwent a CTE or MRE. Dates of colonoscopy and imaging were then matched to identify a cohort who underwent both ileo-colonoscopy and imaging within 6 months of each other. The clinical, radiographic, and endoscopic data were subsequently extracted from the patients’ electronic medical records. For all eligible patients, the following criteria were extracted: (1) Patient demographics (*age*, *gender*, and *smoking status*); (2) CD-related characteristics, including disease duration at the time of cross-sectional imaging, disease location, behavior, extent, and perianal involvement using the Montreal classification^14^; (3) Previous intestinal surgeries, prior and concomitant medical therapy for CD [5-aminosalicyclic acid, corticosteroids, immunomodulators (thiopurines or Methotrexate), biologic or small molecule therapy] at the time of cross-sectional imaging were recorded. Laboratory variables within 3 months of the CTE or MRE were also extracted, including white blood cell (WBC) count, hemoglobin (Hb) count, albumin, and C-reactive protein (CRP).

### Image Acquisition

Computerized tomography enterography and MRE examinations were performed according to local standard techniques.^15-17^ The choice between CTE and MRE was guided by criteria including imaging speed, patient body habitus, access to MRE, and the need to avoid ionizing radiation. Magnetic resonance enterography was generally preferred for initial diagnostics, especially in younger patients, while CTE was used for urgent evaluations. Patients ingested a negative oral contrast consisting of 500-750 mL of lactulose and water solution over 45-60 minutes. For MRE, an additional ingestion of 250 mL of water or Metamucil was ingested 15 minutes prior to imaging. Computerized tomography enterography imaging was acquired in the enteric phase of enhancement at 45 seconds after administration of 100 cc iodinated contrast media intravenously (Omnipaque 350, GE Healthcare). The reconstruction of multiplanar images was performed with a high spatial resolution (slice thickness ≤3 mm) in axial, coronal, and sagittal planes. Magnetic resonance enterography was performed on a 1.5-T magnetic resonance imaging using an 8-channel phased array body coil with gadolinium and an antispasmodic agent (Buscopan). Fast imaging using steady-state acquisition or true fast imaging with steady-state precession images was acquired prior to the administration of 20 mg Buscopan intravenously. After the Buscopan injection, axial and coronal single-shot fast spin-echo T2-weighted images and coronal fat-saturated T2-weighted images were obtained. Coronal images were obtained during dynamic IV gadolinium (Gadovist, Bayer) enhancement (with 3-dimensional volume acceleration [liver acquisition with volume acceleration (LAVA) or volumetric interpolated breath-hold examination (VIBE)] sequences obtained at 45 seconds after the gadolinium administration, with 4 contrast-enhanced phases). In addition, postgadolinium axial LAVA or VIBE images were obtained.

### Image Evaluation

**Figure 1.**
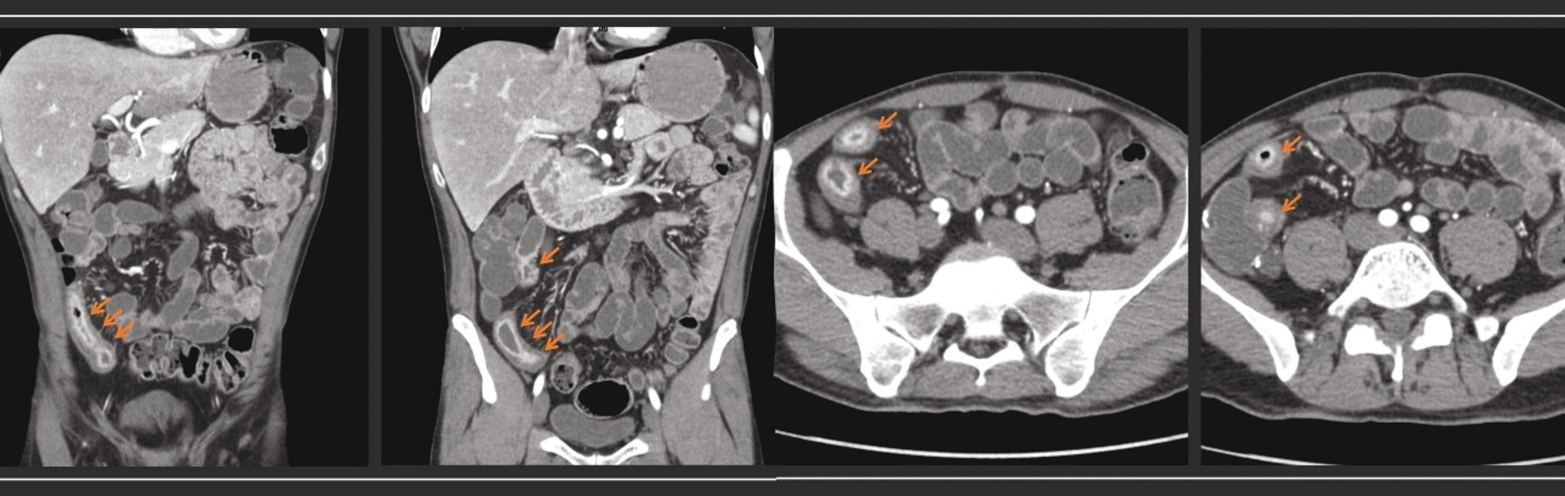
Computerized tomography enterography revealing skip lesions defined as the discontinuous pattern of inflammation along the gastrointestinal tract in distal ileum with areas of luminal stenosis but no upstream dilatation in coronal (A) and axial sections (B).

Radiology reports of all available CTEs and MREs conducted during the time period were manually screened to identify whether the patient had a diagnosis of CD. Once CD had been confirmed, the reports were reviewed for the presence or absence of radiological features suggestive of small bowel inflammation, strictures, or fistulae. The radiological criteria used to define small bowel inflammation included 1 or more of the following components: (1) mural hyperenhancement, (2) mural thickening, (3) mural/mucosal ulceration(s), (4) intramural edema, (5) mesenteric fat inflammation (increased fat attenuation or T2 signal intensity), and (6) the Comb sign (hyperemia and engorgement of the vasa recta).^11,18-20^ Skip lesions were defined as the discontinuous pattern of inflammation along the gastrointestinal tract identified on cross-sectional imaging (Figure 1).

Other radiological data evaluated the presence or absence of a stricture in the small bowel segment. Small bowel strictures were defined based on the following CONSTRICT criteria: (1) mural thickening of at least 25% compared with the normal bowel wall; (2) luminal narrowing <50% of a normal small bowel loop, and (3) proximal small bowel dilation of ≥3 cm on CTE or MRE.^21^ If a stricture was identified, the presence or absence of small bowel inflammation was determined. The presence of penetrating disease-related complications was screened by identifying an abscess or a fistula. If a fistula was identified, it was further characterized as either enteroenteric, enterocolonic, enterovesical, colocolic, perianal, or rectovaginal.

### Outcomes

The primary outcome of our study was the prevalence of endoscopic skipping, defined as a normal ileum on colonoscopy but proximal small bowel inflammation. Secondary outcomes included the prevalence of penetrating complications on cross-sectional imaging complications, including strictures, abscesses, and fistulae, and the prevalence of skip lesions, defined as discontinuous inflammation along the gastrointestinal tract identified on cross-sectional imaging. We also examined specific factors related to patients with CD based on the status of the endoscopic TI (normal vs abnormal).

### Ethical Considerations

Ethical approval for medical chart review was granted by the Western University Research Ethics Board through a delegated and full board review, providing waiver of patient consent.

### Statistical Analysis

Statistical analysis involved summarizing patient and clinical characteristics at baseline using standard descriptive statistics, with mean (SD) for continuous variables and frequency (%) for categorical variables. To compare patients with CD with a normal and abnormal TI—indicating either active inflammation or stricture of the TI or ileocecal valve—a chi-square test of independence was employed for categorical variables, and a 2-sample *t*-test was used for continuous variables.

We conducted a multivariable logistic regression analysis to explore independent predictors for endoscopic skipping, stricturing, and penetrating complications. This analysis aimed to delineate the distinct characteristics and predictors of this specific subset of CD patients.

All analyses were conducted using Statistical Analysis Software (SAS) version 9 (SAS Institute, Cary, NC, USA).

## Results

### Baseline Demographics

A total of 202 patients with clinically active CD who underwent both an ileo-colonoscopy and a CTE or MRE within a 6-month window were eligible for inclusion. The baseline demographics are presented in Table 1. The mean age was 45.2 years (SD 0.49), 39.1% (79/202) were male, and the median disease duration was 9.0 years. Approximately 17.3% (35/202) patients were current smokers and 9.9% (20/202) were previous smokers. Overall, 38.1% (77/202) underwent a previous IBD-related surgery, with 50.6% (39/77) of these patients requiring an ileocolic resection. Approximately 62% (126/202) of patients with CD were maintained on medical therapy, with 34.9% (44/126) on Infliximab, 16.7% (21/126) on Adalimumab, 17.5% (22/126) on Ustekinumab, 11.9% (15/126) on Vedolizumab, 8.73% (11/126) on Methotrexate, and 15.9% (20/126) on Azathioprine/6-Mercaptopurine). 43.1% (87/202) of patients were previously exposed to a corticosteroid, immunomodulator or a biologic. Prior exposure to Methotrexate was identified in 41.4% (36/87) of patients, and 39.1% (34/87) were previously exposed to Infliximab. The mean CRP was 6.42 mg/L (8.7), WBC 6.52 × 10^9^/L (1.9), albumin 41.5 g/L (4.8), and Hb 132.3 g/L (17.9).

**Table 1.** Baseline demographics and disease characteristics within 3 months of the CTE or MRE cross-sectional imaging.

	Total (*N* = 202)
Age (y, mean, SD)	45.2 (0.5)
Male, *n* (%)	79 (39.1)
Smoking history, *n* (%)	
Current, *n* (%)	35 (17.3)
Nonsmoker, *n* (%)	147 (72.8)
Former smoker, *n* (%)	20 (9.9)
Disease duration (y, median, IQR)	9.00 (1-48)
Previous perianal surgery, *n* (%)	
Incision and drainage	9 (4.5)
Seton placement	3 (1.5)
Fistulotomy	9 (4.5)
Previous IBD-related surgery, *n* (%)	77 (38.1)
Small bowel resection	10 (4.9)
Colonic resection	4 (2.0)
Ileocolic resection	39 (19.3)
Subtotal colectomy	6 (3.0)
Hemicolectomy	7 (3.5)
Multiple	10 (5.0)
Current IBD treatment, *n* (%)	126 (62.4)
Corticosteroids (prednisone, budesonide)	2 (1.6)
5-Aminosalicylate	8 (6.3)
Azathioprine/6-Mercaptopurine	20 (15.9)
Methotrexate	11 (8.7)
Infliximab	44 (34.9)
Adalimumab	21 (16.7)
Golimumab	0 (0.00)
Ustekinumab	22 (17.5)
Vedolizumab	15 (11.9)
Combination immunomodulator and biologic agents	2 (1.6)
Clinical trial	0 (0.00)
Previous medication exposure, *n* (%)	87 (43.1)
Corticosteroids (prednisone, budesonide)	2 (2.3)
5-Aminosalicylate	19 (21.8)
Azathioprine/6-Mercaptopurine	24 (27.6)
Methotrexate	36 (41.4)
Infliximab	34 (39.1)
Adalimumab	27 (31.0)
Golimumab	0 (0.00)
Ustekinumab	3 (3.4)
Vedolizumab	5 (5.7)
Other including clinical trial	0 (0.00)
WBC (10^9^/L, mean, SD)	6.52 (1.9)
Hb (g/L, mean, SD)	132.28 (17.9)
Albumin (g/L, mean, SD)	41.50 (4.8)
CRP (mg/L, mean, SD)	6.42 (8.7)

Abbreviations: CRP, C-reactive protein; CTE, computerized tomography enterography; Hb, hemoglobin; IBD, inflammatory bowel disease; IPAA, ileal pouch–anal anastomosis; IQR, interquartile range; MRE, magnetic resonance enterography; WBC, white blood cell.

### Cross-sectional Imaging and Ileo-colonoscopy

Ileo-colonoscopy reports were available in all 202 patients, with 82 (40.6%) undergoing a CTE and 120 (59.4%) MRE. Table 2 summarizes radiographic and endoscopic characteristics. For the primary outcome, the report of endoscopic skipping (ie, small bowel lesions detected on cross-sectional imaging beyond the reach of ileo-colonoscopy) was observed in 22.3% of cases (45/202). Cross-sectional imaging identified active small bowel inflammation in 57.4% (116/202) of patients, utilizing either CTE or MRE. Of these, 82.8% (96/116) were characterized by mural thickening, and 63.8% (74/116) were characterized by mural hyperenhancement. Among all 202 patients who underwent cross-sectional imaging, 24.7% (50/202) had evidence of discontinuous inflammation in the small bowel, that is, skip lesions. These skip lesions were identified in various locations on imaging, with 1.5% (3/202) in the duodenum, 2.5% in the jejunum (5/202), and 20.8% in the ileum (42/202). A normal TI on ileo-colonoscopy was visualized in 40.1% (81/202) of patients.

**Table 2.** Radiological and endoscopic characteristics of patients with CD.

	Total (*n* = 202)
Baseline cross-sectional imaging, *n* (%)	
CTE	82 (40.6)
MRE	120 (59.4)
Small bowel CD proximal to TI intubation, *n* (%)	45 (22.3)
Active radiological inflammation, *n* (%)	116 (57.4)
Mural hyperenhancement	74 (36.6)
Mural thickening	96 (47.5)
Mesenteric fat inflammation	8 (3.7)
Mural ulceration(s)	3 (1.5)
Intramural edema	1 (0.5)
Comb sign	1 (0.5)
Skip lesions on imaging and location, *n* (%)	50 (24.7)
Duodenum	3 (1.5)
Jejunum	5 (2.5)
Ileum	42 (20.8)
Normal TI on ileo-colonoscopy, *n* (%)	81 (40.1)

Abbreviations: CD, Crohn’s disease; CTE, computerized tomography enterography; MRE, magnetic resonance enterography; TI, terminal ileum.

The detection of strictures, assessed through imaging (CTE or MRE), ileo-colonoscopy, or both, revealed a total of 58 cases of stricturing disease (28.7%) (Table 3). Among these cases, 36.2% (21/58) were exclusively detected through cross-sectional imaging (CTE or MRE), 5.1% (3/58) were identified solely via ileo-colonoscopy, and 58.6% (34/58) were diagnosed through both imaging modalities. Regarding the type of strictures observed through cross-sectional imaging, 65.5% (38/55) were characterized by the presence of inflammation, while 34.5% (20/55) exhibited strictures without inflammation. Additionally, 21.8% (12/55) of cases presented with multiple strictures.

**Table 3.** Characteristics and distribution of stricturing disease in Crohn’s patients: imaging, colonoscopy, and stricture types.

	*n*/*N* (%)
Cases with stricturing disease, *n* (%)	58/202 (28.7%)
Detected with only imaging (CTE or MRE)	21/58 (36.2%)
Detected with only ileo-colonoscopy	3/58 (5.1%)
Detected with both	34/58 (58.6%)
Type of stricture based on cross-sectional imaging	
Stricture with inflammation	38/55 (65.5)
Stricture without inflammation	20/55 (34.5)
Multiple stricture	12/55 (21.8)
Stricture with enteric fistula	0 (0.00)

Abbreviations: CTE, computerized tomography enterography; MRE, magnetic resonance enterography.

Table 4 outlines the nature of penetrating disease identified through cross-sectional imaging in patients with CD. Among the 202 patients with CD, abscesses were observed in 2.5% (5/202), while fistulas were in 4.9% (10/202). Enterocutaneous complications were found in 1.0% (2/202), and enteroenteric involvement was noted in 0.5% (1/202). Enterocolonic complications were present in 2% (4/202). Colocutaneous, enterovesical, rectovaginal, and perianal complications were not identified. Multiple fistula types were detected in 3 patients (1.5%).

**Table 4.** Penetrating disease detected by cross-sectional imaging in patients with CD.

	*n*/*N* (%)
Abscess, *n* (%)	5/202 (2.5)
Fistula and type, *n* (%)	10/202 (4.9)
Enterocutaneous	2/202 (1.00)
Enteroenteric	1/202 (0.5)
Enterocolonic	4/202 (2.0)
Colocutaneous	0/202 (0.00)
Enterovesical	0/202 (0.00)
Rectovaginal	0/202 (0.00)
Perianal	0/202 (0.00)
Multiple fistula types	3/202 (1.5)

*n* = number of penetrating complications of interest; *N* = number of patients with CD in the cohort. Abbreviation: CD, Crohn’s disease.

Multivariable logistic regression analyses were conducted for 3 binary response variables: endoscopic skipping, strictures, and penetrating complications. Seven explanatory variables were considered for inclusion in the models: sex, age, smoking status, disease duration, previous therapy, perianal surgery, and previous resection. At a significance of 10% level, there was no evidence to suggest any of the factors were associated with endoscopic skipping. Disease duration (*P* = .08) and perianal surgery (*P* = .06) were found to be indicatively associated with stricture, while age and disease duration were associated with penetration complications (Supplementary Table).

### Characteristics of Patients With Active CD With Radiographic Small Bowel Inflammation but Normal TI

Results in Table 5 compare clinical characteristics between 2 groups of patients with active CD, specifically focusing on those with a normal TI on colonoscopy (*n* = 81) and those with an abnormal TI (*n* = 121). There were no significant differences between the median ages, gender, smoking status, previous resection, or disease duration. However, previous perianal surgery was more prevalent in the group with an abnormal TI on ileo-colonoscopy (6.6%) compared with the group with normal TI (16.0%) on ileo-colonoscopy, showing a statistically significant difference (*P* = .03). Conversely, previous resection rates did not significantly differ between the 2 groups (*P* = .80). C-reactive protein levels were significantly higher in the group with abnormal TI compared with the group with normal TI (4.31 [SD 5.17] vs 8.00 [SD 10.43], *P* = .005).

**Table 5.** Comparison between patients who had active Crohn’s disease with and without abnormal TI on colonoscopy.

Clinical characteristics	Active Crohn’s disease and normal TI (*n* = 81)	Active Crohn’s disease and abnormal TI (*n* = 121)	*P* value
Age (y, median [range])	44.00 (15.91)	46.00 (16.55)	.88
Sex			
Male	34 (41.98)	45 (37.19)	.50
Female	47 (58.02)	76 (62.81)	
Smoking history, *n* (%)			
Former smoker	7 (8.64)	13 (10.74)	.50
Smoker	17 (20.99)	18 (14.88)	
Nonsmoker	57 (70.37)	90 (74.38)	
Duration of Crohn’s disease (y)			
≤5	35 (43.21)	49 (40.50)	.74
>5	46 (56.79)	71 (58.68)	
Previous perianal surgery, *n* (%)	13 (16.05)	8 (6.61)	.03
Previous resection, *n* (%)	30 (37.04)	47 (38.84)	.80
C-reactive protein, mean (SD), mg/L	4.31 (5.17)	8.00 (10.43)	.005

Abbreviation: TI, terminal ileum.

## Discussion

The results from this study provide important insights into the diagnostic challenges of small bowel CD. Despite ileo-colonoscopy reports being available for all patients, almost 25% exhibited evidence of endoscopic skipping proximal to the TI intubation. Moreover, over a third of the cases with stricturing disease would be missed without the use of cross-sectional imaging. The discrepancy between ileo-colonoscopy and radiographic inflammation could be secondary to noncontiguous skip lesions proximal to the distal TI, intramural, transmural, or mesenteric inflammation identifiable on imaging only.

Our findings are consistent with prior smaller reports. Siddiki et al conducted a prospective study with 33 patients who underwent CTE or MRE and ileo-colonoscopy.^11^ Cross-sectional imaging identified 8 cases (24%) with active small bowel inflammation in which ileocolic examination was normal.^11^ Similarly, Samuel et al conducted a retrospective cohort study of 153 patients with CD at the Mayo Clinic, in which 67 (43.8%) had a macroscopically normal TI despite 36 of these patients (53.7%) having active small bowel imaging on CTE or MRE.^12^ Data from pediatric patients with small bowel CD had shown endoscopic TI skipping was present in 59% of patients.^13^

Obtaining a baseline MRE or CTE for staging as an adjunct to ileo-colonoscopy is noteworthy.^22^ This approach recognizes the limitations of conventional endoscopic techniques, especially in evaluating proximal small bowel inflammation. By establishing a baseline through MRE or CTE, clinicians can gain a comprehensive understanding of the disease distribution, enabling more accurate interpretation of subsequent imaging modalities. This approach is important for accurate diagnosis and subsequent management of CD to assess response to therapy, particularly in cases where traditional endoscopic examinations may yield normal results despite active disease. The observed association between elevated CRP levels and abnormal TI emphasizes the potential role of inflammatory markers in refining diagnostic strategies for CD and subsequent treatment plans.

This study also highlights the importance of cross-sectional imaging in the detection of stricturing and penetrating complications. If cross-sectional imaging (CTE or MRE) were not performed, at least a third of the cases with stricturing disease in CD would go undetected. Specifically, of the total 58 cases with stricturing disease, 21 cases (36.2%) would have been missed by relying solely on ileo-colonoscopy, and an additional 16 cases (27.6%) would have not been identified without the combined approach of both imaging and ileo-colonoscopy. Therefore, the absence of cross-sectional imaging would result in the oversight of a substantial proportion (36.2%) of stricturing cases in this cohort. We also found that 7.4% of patients experienced various penetrating complications, including abscesses and different types of fistulae that were only detected on imaging.

Aside from cross-sectional imaging with CTE and MRE, there is growing interest in intestinal ultrasound (IUS) as a noninvasive evaluative tool for small bowel inflammation.^23,24^ By utilizing a combination of imaging modalities, for example, including MRE or CTE for initial staging for a comprehensive small bowel assessment and then IUS for ongoing assessments, clinicians can address the challenges associated with residual symptoms and ensure a more accurate and nuanced evaluation of CD.^7^ CTE is less dependent on body habitus, quicker, and less prone to artifacts than MRE and IUS; nevertheless, because it employs ionizing radiation, it may be less appropriate for the serial exams often needed in the management of IBD. To note, dose-reducing techniques are continuously being developed to mitigate the potential risks while still maintaining diagnostic accuracy. These techniques include advancements such as multienergy CT, photon counting CT, and low-dose single-energy protocols. Although prospective multicenter head-to-head comparisons suggest MRE is more accurate, especially for defining the extent of small bowel CD,^25^ and probably preferable for the initial diagnostic workup to define the disease distribution and phenotype, its use can be limited by access in some healthcare systems.

By comparing clinical features between individuals with clinically active CD and a normal TI vs those with an abnormal TI, we aimed to identify specific features that might be associated with clinical activity despite endoscopic findings suggesting normality in the TI. Our analysis did not find significant differences in baseline characteristics such as median age, gender, smoking status, previous resection, or disease duration between patients with CD with a normal and abnormal TI. However, there was a notable distinction in CRP levels, with higher values observed in those with an endoscopically abnormal TI compared with those with a normal TI. The elevated CRP in patients with abnormal TI underscores the utility of this inflammatory marker as an adjunctive diagnostic tool and the merit of cross-sectional imaging for confirmation of the status of disease activity and subsequent therapeutic plans. Prior studies have shown that patients with clinically active CD but normal TI on colonoscopy also had a higher prevalence of perianal disease.^12^ Both perianal disease and the perforating phenotype serve as indicators of aggressive CD. Whether patients exhibiting endoscopic skipping represent a more aggressive phenotype requires further examination in a larger study cohort. Importantly, we found that the prior history of perianal surgery was notably greater among individuals with a normal TI on colonoscopy compared with those with an abnormal TI (16.05% vs 6.61%, *P* = .03).

Our study has several strengths when compared with prior published work. First, to our knowledge, this is the largest cohort study published to date examining the prevalence of endoscopic skipping, and is larger than an earlier study from the Mayo Clinic.^12^ Second, our results are strengthened by the reliability of endoscopic and radiologic assessments being performed by the same endoscopists and fellowship-trained radiologists following the same hospital protocol and blinded to the colonoscopy results. The limitations of this study include its retrospective design and the inherent selection bias stemming from the recruitment of patients from a tertiary academic center. Additionally, the study lacks data on esophago-gastro-duodenoscopy and histopathology, which could provide a more comprehensive assessment of the disease. Consequently, the findings may not be applicable to population-based cohorts, limiting the generalizability of the results.

The results of this study and previously published data demonstrate that small bowel CD may be detected at cross-sectional enterography despite normal ileo-colonoscopic findings in adult patients, in approximately 25% of cases. Computerized tomography enterography or MRE is complementary to upper endoscopy or ileo-colonoscopy for the diagnosis, management, and assessment of response to treatment for CD.

## Supplementary Data

Supplementary data is available at *Inflammatory Bowel Diseases* online.

izae192_suppl_Supplementary_Table
