# Correction to: Endoscopic Skipping, Stricturing, and Penetrating Complications in Crohn’s Disease on Tandem Ileo-colonoscopy and Cross-sectional Imaging: A Retrospective Cohort Study

**DOI:** 10.1093/ibd/izae246

**Published:** 2024-10-01

This is a correction to: Virginia Solitano, Sudheer Kumar Vuyyuru, Achuthan Aruljothy, Maan Alkhattabi, Joshua Zou, Melanie Beaton, Jamie Gregor, Zahra Kassam, Rocio Sedano, Harry Marshall, Darryl Ramsewak, Michael Sey, Vipul Jairath, Endoscopic Skipping, Stricturing, and Penetrating Complications in Crohn’s Disease on Tandem Ileo-colonoscopy and Cross-sectional Imaging: A Retrospective Cohort Study, *Inflammatory Bowel Diseases*, 2024;, izae192, https://doi.org/10.1093/ibd/izae192

In the version originally published, the fifth author was incorrectly associated with the following department:

Department of Biostatistics, University of Waterloo, Waterloo, Ontario, Canada

This has been corrected to read:

Department of Statistics and Actuarial Science, University of Waterloo, Waterloo, Ontario, Canada.

In addition, the term ‘structuring’ in the final sentence of the ‘Methods’ section in the article abstract has been corrected to read ‘stricturing’.

