# Supplementary material for: Endoscopic Skipping, Stricturing, and Penetrating Complications in Crohn’s Disease on Tandem Ileo-colonoscopy and Cross-sectional Imaging: A Retrospective Cohort Study
Source: Inflamm Bowel Dis. 2024 Aug 31;31(6):1529–36. doi: 10.1093/ibd/izae192 (PMC12166302; doi:10.1093/ibd/izae192)
Supplement: izae192_suppl_Supplementary_Table [file izae192_suppl_supplementary_table.docx]

Supplementary Table. Results of multivariable logistic regression analyses for endoscopic skipping, strictures, and penetration complications

|  | Skipping lesions | | | | Strictures | | | | Penetration Complications | | | |
| --- | --- | --- | --- | --- | --- | --- | --- | --- | --- | --- | --- | --- |
| Factor | OR | 95% CI | | p-value | OR | 95% CI | | p-value | OR | 95% CI | | p-value |
| Sex | 1.27 | 0.63 | 2.56 | 0.51 | 1.68 | 0.87 | 3.26 | 0.12 | 1.25 | 0.36 | 4.38 | 0.72 |
| Age | 0.99 | 0.97 | 1.01 | 0.31 | 1.00 | 0.98 | 1.03 | 0.84 | 0.91 | 0.84 | 0.99 | 0.02 |
| Smoking 0 vs 2 | 1.60 | 0.41 | 6.28 | 0.35 | 4.10 | 0.84 | 19.97 | 0.10 | 0.19 | 0.03 | 1.27 | 0.16 |
| 1 vs 2 | 1.09 | 0.23 | 5.20 | 0.79 | 3.30 | 0.59 | 18.32 | 0.39 | 0.30 | 0.04 | 2.60 | 0.67 |
| Disease Duration | 1.00 | 1.00 | 1.01 | 0.54 | 1.03 | 1.00 | 1.07 | 0.08 | 1.09 | 0.99 | 1.20 | 0.07 |
| Previous therapy | 1.82 | 0.85 | 3.92 | 0.13 | 0.67 | 0.32 | 1.43 | 0.30 | 2.95 | 0.66 | 13.16 | 0.17 |
| Perianal surgery | 0.35 | 0.08 | 1.65 | 0.19 | 0.22 | 0.05 | 1.05 | 0.06 | 1.11 | 0.19 | 6.45 | 0.91 |
| Previousresection | 0.60 | 0.26 | 1.37 | 0.23 | 1.17 | 0.50 | 2.76 | 0.72 | 0.77 | 0.15 | 3.83 | 0.75 |
